# Simple Detection and Culture of Circulating Tumor Cells from Colorectal Cancer Patients Using Poly(2-Methoxyethyl Acrylate)-Coated Plates

**DOI:** 10.3390/ijms24043949

**Published:** 2023-02-16

**Authors:** Masatoshi Nomura, Yuhki Yokoyama, Daishi Yoshimura, Yasuhisa Minagawa, Aki Yamamoto, Yukiko Tanaka, Naoko Sekiguchi, Daiki Marukawa, Momoko Ichihara, Hiroaki Itakura, Kenichi Matsumoto, Yoshihiro Morimoto, Hideo Tomihara, Akira Inoue, Takayuki Ogino, Norikatsu Miyoshi, Hidekazu Takahashi, Hidenori Takahashi, Mamoru Uemura, Shogo Kobayashi, Tsunekazu Mizushima, Takahisa Anada, Masaki Mori, Yuichiro Doki, Masaru Tanaka, Hidetoshi Eguchi, Hirofumi Yamamoto

**Affiliations:** 1 Department of Gastroenterological Surgery, Graduate School of Medicine, Osaka University, Suita 565-0871, Japan; 2 Department of Molecular Pathology, Division of Health Sciences, Graduate School of Medicine, Osaka University, Suita 565-0871, Japan; 3 Department of Planning & Administration, Headquarters of Research & Development, Sumitomo Rubber Industries, Ltd., Kobe 651-0071, Japan; 4 Institute for Materials Chemistry and Engineering, Kyushu University, Fukuoka 819-0395, Japan; 5 Department of Gastroenterological Surgery, Osaka General Medical Center, Osaka 558-8558, Japan; 6 Department of Gastroenterological Surgery, Osaka Police Hospital, Osaka 543-0035, Japan; 7 Graduate School of Medicine, Tokai University, Isehara 259-1193, Japan

**Keywords:** CTC, colorectal cancer, PMEA, cell culture, spheroid

## Abstract

Here we aimed to establish a simple detection method for detecting circulating tumor cells (CTCs) in the blood sample of colorectal cancer (CRC) patients using poly(2-methoxyethyl acrylate) (PMEA)-coated plates. Adhesion test and spike test using CRC cell lines assured efficacy of PMEA coating. A total of 41 patients with pathological stage II–IV CRC were enrolled between January 2018 and September 2022. Blood samples were concentrated by centrifugation by the OncoQuick tube, and then incubated overnight on PMEA-coated chamber slides. The next day, cell culture and immunocytochemistry with anti-EpCAM antibody were performed. Adhesion tests revealed good attachment of CRCs to PMEA-coated plates. Spike tests indicated that ~75% of CRCs from a 10-mL blood sample were recovered on the slides. By cytological examination, CTCs were identified in 18/41 CRC cases (43.9%). In cell cultures, spheroid-like structures or tumor-cell clusters were found in 18/33 tested cases (54.5%). Overall, CTCs and/or growing circulating tumor cells were found in 23/41 CRC cases (56.0%). History of chemotherapy or radiation was significantly negatively correlated with CTC detection (*p* = 0.02). In summary, we successfully captured CTCs from CRC patients using the unique biomaterial PMEA. Cultured tumor cells will provide important and timely information regarding the molecular basis of CTCs.

## 1. Introduction

Colorectal cancer (CRC) is one of the most prevalent cancers worldwide, and its incidence has increased over recent years. In 2018, CRC was responsible for the second and third highest rates of mortality (9.2%) and morbidity (10.2%), respectively [1]. Despite advances in therapeutic options, the 5-year survival rate remains <65%, and approximately 90% of cancer deaths are caused by cancer metastasis [2,3]. To improve the prognosis of CRC patients, it is essential to detect tumor cells in a timely manner. Tumor samples are usually obtained by preoperative biopsy or surgical resection, but tumor cell sampling can be difficult in patients with advanced disease, particularly when the tumor is located deep in the body.

In recent years, research attention has focused on liquid biopsy, which enables the timely collection of circulating tumor DNA (ctDNA) at any time-point [4]. Several key genes, such as *EGFR*, *KRAS*, and *BRAF*, and microsatellite instability (MSI), are detectable in blood samples [5]. Knowledge of mutation status can guide the selection of appropriate therapeutic options [6]. Circulating tumor cells (CTCs) are an alternative detection target in liquid biopsy. The number of CTCs is significantly correlated with prognosis in carcinomas of the colorectum, prostate, and breast [7,8,9,10]. Unlike ctDNA, CTC analysis provides information on RNA expression [11,12], in addition to gene alterations, and certain CTCs from prostate cancer patients produce organoids [13]. Therefore, CTC analysis has broad applications in terms of in vitro and in vivo biological analyses for tumor characteristics, which may enable assessment of drug sensitivity and prediction of recurrence [14,15].

In the CellSearch system, antibody-dependent cell capture is used to collect epithelial marker EpCAM-positive and leukocyte marker CD45-negative cells as CTCs [16]. On the other hand, several CTC collection methods have been developed based on physical property of tumor cells [17], including single-spiral microchannel, Ficoll gradient, filtration, and chip principle methods. However, with all systems, a common dilemma is encountered during the process of enriching and isolating CTCs from the blood—as more CTCs are gathered, more blood cells are mixed in. On the other hand, prioritization of CTC purity results in greater loss of CTCs. In CRC, the concentration of CTCs is rather low—with fewer than 5–10 CRCs in 10 mL of peripheral blood from most cases [18,19,20,21]. This makes it very difficult to initiate cell culture using CTCs from CRC, and increases the importance of collecting only CTCs while excluding blood cells as much as possible. 

Recent advances in biomedical engineering have led to the development of an antibody-free polymeric biomaterial, poly(2-methoxyethyl acrylate) (PMEA). PMEA has been approved by the FDA for use in medical devices, and PMEA has the largest market share in the world as a nonthrombogenic coating agent for artificial oxygenators. Our team has demonstrated by in vitro studies that breast cancer and other tumor cells can attach to this blood-compatible PMEA via both integrin-dependent and -independent mechanisms [22,23,24,25,26,27], while platelets and other blood cells cannot attach to PMEA [28,29,30,31,32,33]. 

In the present study, we used PMEA-coated plates to capture scarce CTCs from blood samples obtained from CRC patients. To detect CTCs, we established optimal immunostaining conditions for the EpCAM epithelial marker on PMEA-coated plates. We also attempted to culture CTCs for possible applications in DNA and RNA analyses. To our knowledge, this is the first study of the clinical application of PMEA coating for efficient isolation of CTCs from blood samples of cancer patients. 

## 2. Results

### 2.1. Adhesion of CRC Cell Lines to PMEA-Coated and Non-Coated Plates

**Figure 1:**
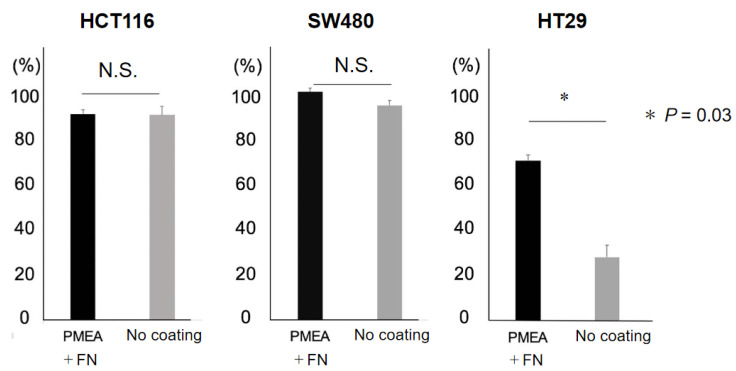
Adhesion test. After labeling colorectal cancer (CRC) cell lines with CellTracker, 100 CRC cells were seeded on plates coated with PMEA plus fibronectin or non-coated plates. Cells were incubated in standard medium overnight, and the number of attached cells was counted under a fluorescence microscope. HCT116 and SW480 cells attached well to the PMEA + fibronectin-coated plates. HT29 cells showed a significantly greater rate of attachment to PMEA + fibronectin-coated plates (69.3% ± 4.7%) compared to non-coated plates (27.5% ± 8.7%) (* *p* = 0.03). FN, fibronectin; N.S., not significant.

The CRC cell lines were labeled with the CellTracker, and then 100 CRC cells were seeded on PMEA/fibronectin-coated plates or non-coated plates. Cells were incubated in standard medium overnight, and the number of attached cells was counted under a fluorescence microscope. The majority of HCT116 and SW480 cells attached well to both PMEA/fibronectin-coated and non-coated plates, while the HT29 cells only showed <30% attachment to non-coated plates (Figure 1). Notably, HT29 cells showed a significantly greater attachment rate to PMEA/fibronectin-coated plates (69.3% ± 4.7%) compared with non-coated plates (27.5% ± 8.7%) (*p* = 0.03; Figure 1). 

### 2.2. Spike Test

**Figure 2:**
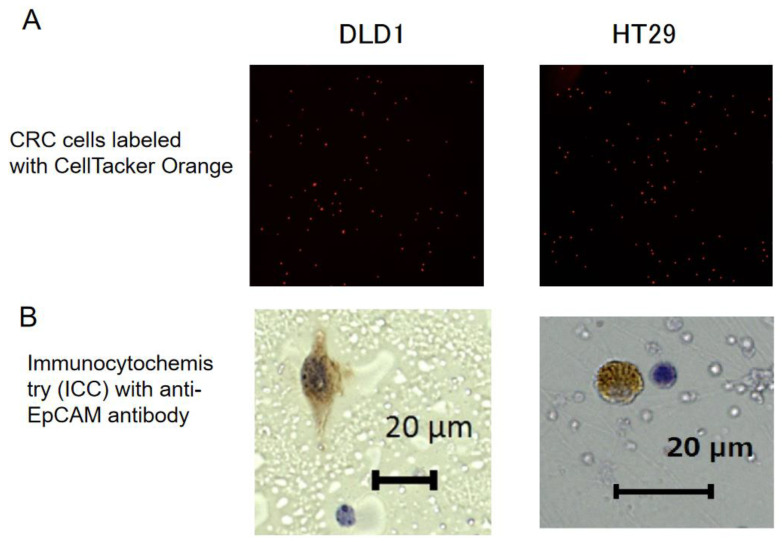
Spike test. (**A**) Cells were labeled with CellTracker Orange, and all cells were well marked. After enrichment using the OncoQuick tube, the cell recovery rate from 10 mL blood samples was 89.2% for HT29, and 90.9% for DLD-1. (**B**) Immunocytochemistry for EpCAM. Colorectal cancer (CRC) cells showed positive staining in brown, and were larger than nearby leukocytes. Counter-staining was performed with hematoxylin solution. Scale bar: 20 μm.

To count the cell number, HT29 and DLD-1 cells were labeled with the CellTracker, and all cells were well-marked with orange fluorescence (Figure 2A). We made a cell suspension containing 148 HT29 cells and 121 DLD-1 cells per 10 μL, and mixed 50 μL of this cell suspension with 10 mL of venous peripheral blood from healthy donors. The mixture was added to an OncoQuick tube (Supplementary Figure S1). After centrifugation, the recovered cells included 132 HT29 cells (recovery rate 89.2%) and 110 DLD-1 cells (recovery rate 90.9%). These recovered cells (132 HT29 and 110 DLD-1 cells) were seeded on PMEA/fibronectin-coated slides. The next day, immunostaining was performed using anti-EpCAM antibody (Figure 2B). EpCAM is supposed to be expressed in 93.6% HT29 cells and 84.8% DLD1 cells [34]. EpCAM was expressed by 98 and 103 HT29 cells, and by 87 and 83 DLD-1 cells, indicating that the recovery rate should exceed 75% throughout the procedure (Supplementary Table S1). 

### 2.3. Immunocytochemistry for EpCAM and Cytological Examination

**Figure 3:**
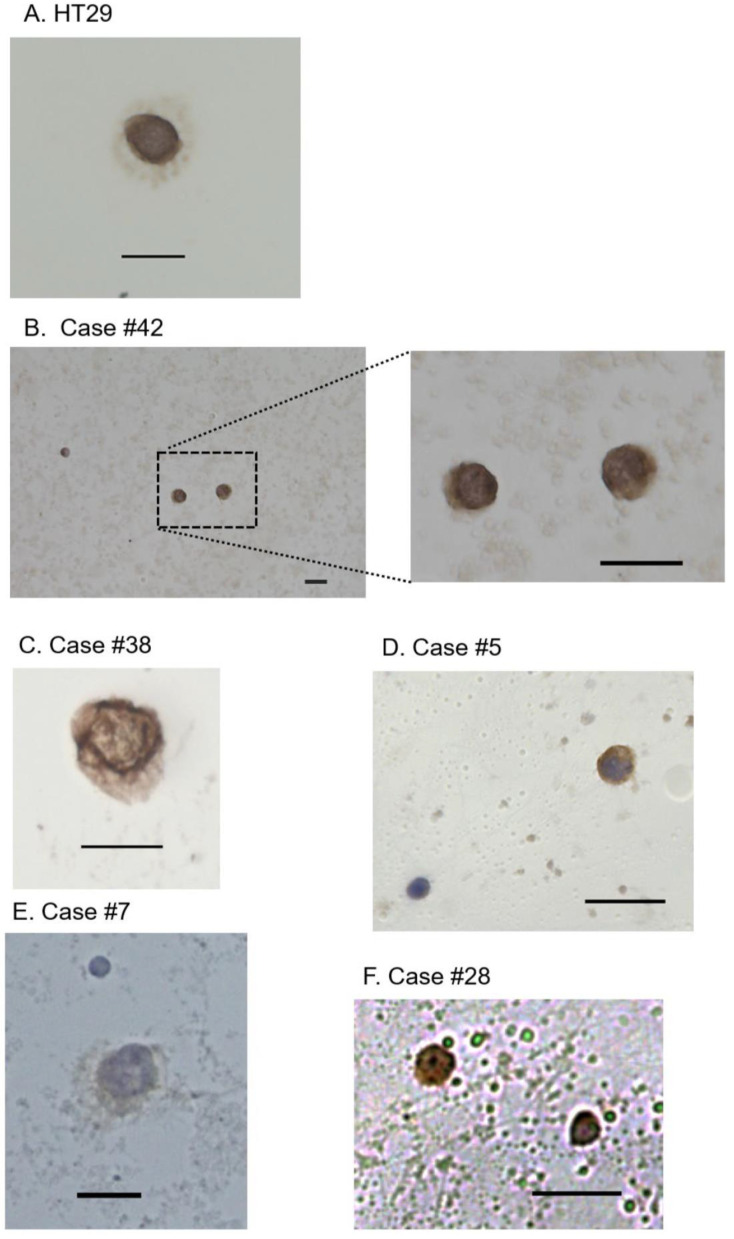
Immunocytochemistry for EpCAM and cytological examination of circulating tumor cells (CTCs.) (**A**) An HT29 cell. This cell line served as a positive control for EpCAM staining. (**B**) EpCAM-positive CTCs in a case of stage III rectal cancer with bladder invasion (Case #42). (**C**) An EpCAM-positive CTC in a case of stage IV sigmoid colon cancer with pulmonary and hepatic metastases (Case #38). (**D**) An EpCAM-positive CTC in a case of stage IV rectosigmoid colon cancer with hepatic metastasis (Case #5). A leukocyte in the bottom left corner was not stained with anti- EpCAM antibody. (**E**) An EpCAM-negative CTC in a case of stage IV sigmoid colon cancer with lung metastasis (Case #7). A large-sized, irregular-shaped nucleus was evident, as compared to a leukocyte located in the upper side. A nucleolus was also notable. (**F**) EpCAM-positive CTCs in a case of stage IV ascending colon cancer with metastases to the liver, lung, and peritoneum (Case #28). Scale bars: 20 μm.

Table 1 shows the background information for the 41 CRC patients enrolled in this study. The majority (38/41, 92.7%) had stage IV disease. From 10 mL peripheral venous blood, we found that CTCs were captured for 18 of 41 CRC patients (43.9%) based on cytological diagnosis. Using HT29 cells as a positive control (Figure 3A), 16 of 18 cases had EpCAM-positive CTCs (representatively shown in Figure 3B–D,F), and two cases had EpCAM-negative CTCs (one example shown in Figure 3E). CTCs exhibited an enlarged and irregular-shaped nucleus, and had an increased N/C ratio (Figure 3B–F). Some CTCs exhibited a nucleolus (Figure 3E,F). We examined the relationship between CTC detection and clinicopathological parameters, and found that CTC detection was significantly associated with histology of mucinous or poorly-differentiated adenocarcinoma (*p* = 0.02, Supplementary Table S2, although histological type was not identified in 7 of 41 patients in whom primary tumor resection was not possible.

### 2.4. Cell Culture

**Figure 4:**
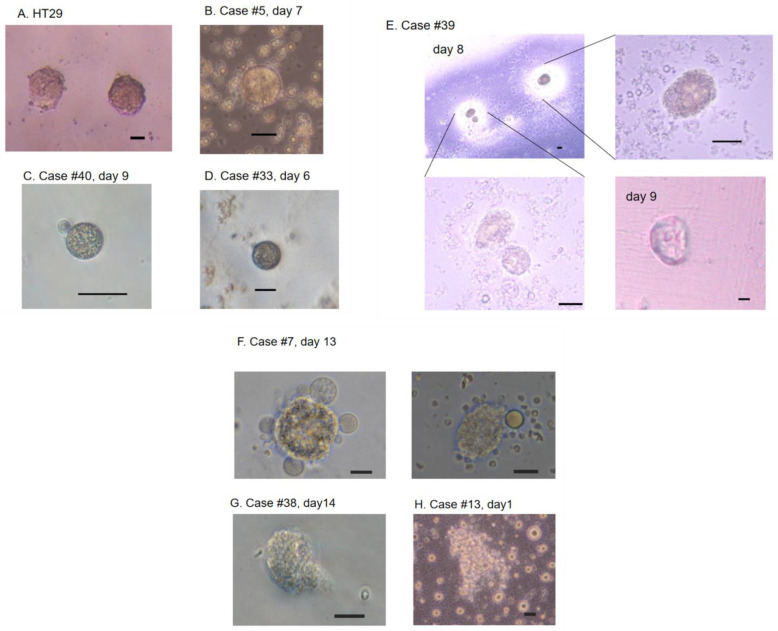
Cell culture of circulating tumor cells (CTCs). (**A**) Spheroid-like appearance of HT29 cells. When HT29 cells were cultured on Matrigel-coated plates, they grew to show a spheroid-like structure on day 10. (**B**) A spheroid-like cell appeared on day 7 in Case #5. (**C**) A spheroid-like cell appeared on day 9 in Case #40. (**D**) A spheroid-like cell appeared on day 6 in Case #33. (**E**) Spheroid-like cells appeared on day 8 in Case #39. Magnified views are also shown. On day 9, a large spheroid-like structure appeared. (**F**) Spheroid-like cells appeared on day 13 in Case #7. (**G**) A spheroid-like cell appeared on day 14 in Case #38. (**H**) A large cluster comprising many tumor cells was visible on day 1 in Case #13. Scale bars: 20 μm.

After establishment of CTC detection, we started cell culture from 35 CRC cases. Two cases were excluded due to contamination or gelatinization of the coating material. As a positive control for cell culture, HT29 cells were cultured on Matrigel with the ES medium. These HT29 cells exhibited a spheroid-like appearance on day 10 (Figure 4A). Among the clinical samples, cell culture was successful in 18 of 33 CRC cases (54.5%, Figure 4B–H). Blood cells, such as leukocytes and erythrocytes, gradually disappeared each day (Supplementary Figure S2). Tumor cells remained and grew to diameters of up to 15–30 μm in 7–9 days (Figure 4B–D), with some exceeding 50 μm on days 9–14 (Figure 4E–G). Most cells showed a spheroid-like morphology (Figure 4B–G). One case exhibited large clusters >100 μm, which comprised many tumor cells (Figure 4H).

Examining the relationship between the presence of growing tumor cells and clinicopathological parameters revealed that history of chemotherapy or radiation was significantly associated with failure of cell culture (*p* = 0.0004, Supplementary Table S3. When combining the positive results from cytological examination and/or cell culture, CTCs were found in 23 of 41 CRC cases (56.1%). We found that failure to detect CTCs by either method was significantly associated with history of previous chemotherapy or radiation (Table 2, *p* = 0.02).

**Figure 5:**
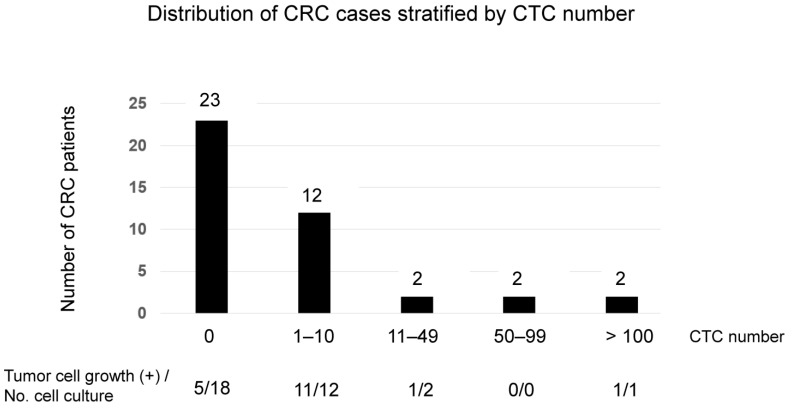
Distribution of CRC cases stratified by CTC number. Distribution of colorectal cancer (CRC) cases when stratified by the number of circulating tumor cells (CTCs) detected by cytology. In the majority of CRC cases (85.4%, 35/41), the detected CTC number was 0 or 1–10. Tumor growth was observed at high incidence (11 of 12 cases) in cases where 1–10 CTCs were detected, and sometimes feasible (5 of 18 cases) in cases where CTC number was 0.

Figure 5 shows the distribution of CRC cases when stratified by the CTC number detected by cytological examination. In the majority of CRC cases (85.4%, 35/41), the detected CTC number was 0 or 1–10. Tumor growth was observed at a high incidence (11 of 12 cases) in cases where 1–10 CTCs were detected, and sometimes feasible (5 of 18 cases) in cases where the CTC number was 0. 

## 3. Discussion

The CellSearch system uses the epithelial-cell marker EpCAM. Currently used CRC cell lines HCT116, SW480, HT29, and DLD-1 express EpCAM at a high incidence, ranging from 82.7 to 100% [34,35]. However, cells with metastatic potential are likely to exhibit mesenchymal characteristics and lose epithelial features [36]. Before our current study, we performed single-cell mutation analysis of the *KRAS*, *BRAF*, and *PIK3CA* genes in CTCs from CRC patients, using dielectrophoresis-based capture in a micro-pore system. *KRAS* or *PIK3CA* mutations were present in the CTCs from 26.7% of the CRC patients, and some cases had mutations in non-epithelial CTCs lacking cytokeratin expression [37]. In the present study, we attempted to capture CTCs by concentration with an OncoQuick tube, followed by additional CTC purification using PMEA/fibronectin-coated chamber slides. This simple system is antibody-free and does not require any special device. Within our current case series, the CTCs from most CRC cases expressed the EpCAM protein. However, two CRC cases exhibited EpCAM-negative CTCs, and both had rather high numbers of CTCs: 20 CTCs in Case #13 (Figure 4H) and >100 CTCs in Case #7 (Figure 3E and Figure 4F). These findings suggest that additional staining of CTCs with a mesenchymal marker, e.g., plastin 3 [38], may improve the detection rate. 

The blood-compatible polymer PMEA has been extensively studied by our team (Prof. Tanaka M and his colleagues) in investigations of “antibody-free” attachment-based cell collection and enrichment [22,23,24,25,26,27]. Optimal PMEA concentration was previously determined [39]. Tanaka M et al. suggest that intermediate water plays a central role in preventing the attachment of platelets to PMEA-coated slides [22,40,41,42]. The amount of intermediate water bound to this polymer regulates the interaction between PMEA and the cell membrane, due to changes in the adsorption behaviors of glycoproteins and proteoglycans [40]. Without specific cell-surface markers, they demonstrated that cancer cell lines—e.g., breast cancer cells, hepatocellular carcinoma cells (HCCs), and fibrosarcoma cells—can adhere to PMEA-coated plates via integrin-dependent or -independent mechanism [25,26,27]. Importantly, studies also show that fibronectin improves the PMEA-mediated adhesion of tumor cells; upon absorption onto the surface of PMEA, fibronectin becomes denatured, which initiates the integrin-mediated adhesion of tumor cells [23,43]. Therefore, in our present study, we added a fibronectin coating onto the PMEA coating.

We previously found that HCC cells were likely to be present in the blood of patients with HCC, because qRT-PCR often revealed mRNA for the HCC-specific marker alpha-fetoprotein (AFP) in blood samples. Specifically, AFP mRNA was detected in 9 of 38 (23.7%) of the peripheral blood samples collected from HCC patients, and the presence of AFP mRNA was significantly correlated with distant metastasis, and shorter disease-free survival [44,45]. Moreover, we were consistently able to capture relatively abundant CTCs from patients with HCC using the PMEA coating method (our unpublished observation, a few examples are shown in Supplementary Figure S3). On the other hand, only small numbers of CTCs are found in the blood of CRC patients, with most CRC cases having 0 or 1–10 CTCs (median or mean value, 4–5) [18,19,20,21]. Therefore, in the present study, we focused on the extent to which such small numbers of CTCs can be captured using PMEA-coated plates, from blood samples of CRC patients. 

Our in vitro adhesion test results indicated that HT29 colon cancer cells exhibited better adhesion to PMEA-coated slides compared to non-coated slides. The spike test revealed that the CRC cell recovery rate should exceed 75%. In our prospective clinical study, we detected CTCs in the blood samples of 23 of 41 advanced CRC patients (56.1%) by cytological examination, and/or growing tumor cells in cell culture. Interestingly, CTCs were detected not only in stage IV CRC cases, but also in one stage II and two stage III CRC cases. Among these three cases, two cases (one stage II and one stage III CRC) exhibited an invasion on the bladder, and the other stage III CRC case exhibited a large (6.0 cm) tumor with an invasion on the lymphatic duct. These data suggest the potential usefulness of PMEA-mediated capture methods to further investigate intermediate stage II or III CRC cases. We found that CTCs were more likely to be detected in patients who did not receive previous chemotherapy or radiation. Therefore, it would be better to perform CTC assessment prior to such therapies.

In our first 15 preliminary test cases (not included in this study), we did not find any CTCs using fluorescein-conjugated EpCAM antibody. We think that this is because of the small numbers of EpCAM-positive CTCs, weak fluorescence signal, and unexpectedly abundant surrounding blood cells, even after concentration with the OncoQuick tube. When using a higher titer of antibody, the background fluorescence signal rendered the detection of CTCs rather difficult. Therefore, we changed the staining method from fluorescence to the peroxidase reaction, which we expected would provide advantages to ensure careful and repeated observation under the light field. When quenching the endogenous peroxidase activity, methanol dissolved the PMEA; therefore, instead of methanol, we used distilled water with 0.1% sodium azide containing 0.3% hydrogen peroxidase, as previously performed [46]. In the initial three CRC cases, we stained CTCs using the avidin-biotin complex (ABC) method. Thereafter, we employed one-step staining using the peroxidase-conjugate primary antibody, to prevent the CTCs from peeling off the slides due to repeated washing with PBS. Eventually, we established a stable immunostaining method.

It has been questioned whether a few CTCs might be expanded enough to enable comprehensive RNA profiling, as well as DNA mutation analysis. It was previously reported that CTC culture was achieved in only 2 of 71 non-treated CRC cases (2.8%), which had >300 CTCs [18]. In contrast, in our present study, cell culture of CTC was successful using samples from 18 of 33 CRC patients (54.5%), despite inclusion of 11 cases that had been previously treated with chemotherapy or radiation. Additionally, we succeeded in generating growing tumor cells at a high rate, even from cases with as few as <10 CTCs (Figure 5). Our highly successful results may be explained by the suggestion that PMEA might promote tumor cell viability, as previously demonstrated [25,26]. Notably, the success rate in cell culture was even higher (54.5%) than that determined by a cytological survey (43.9%). Moreover, even among samples where CTCs were not detected by cytological survey, in 5 of 18 CRC cases, cell culture produced viable spheroid-like structures (Figure 5). This result may be because we assigned more than half a portion of the concentrated cell fraction to cell culture (e.g., 80% versus 20%, Supplementary Figure S1), and possibly because a portion of CTCs might be peeled off during the staining procedure.

We postulate that the proportion of CTCs to blood cells may be quite important in determining the success of generation of growing tumor cells in cell culture. When tumor cells are abundantly present, they are likely to expand irrespective of the coexistence of many leukocytes and erythrocytes. However, if there are few tumor cells, they are unlikely to expand in the presence of numerous blood cells. Using the OncoQuick tube to enrich the CTCs certainly reduced the amounts of erythrocytes and leukocytes. However, considerable numbers of blood cells remained even after treatment. We attempted direct culture on non-coated chamber slides after OncoQuick treatment, and found that although the chamber was initially occupied by numerous erythrocytes and some leukocytes, almost none remained after one week (Supplementary Figure S4). Since this was just one case trial, further investigation is needed. On the other hand, after CTCs and the remaining blood cells were transferred together from the PMEA-coated chamber slide to the culture plate, the remaining blood cells gradually disappeared each day, while sphere-like structures survived (Supplementary Figure S2). Together with the previous evidence that blood cells do not attach to PMEA [28,29,30,31,32], these findings suggest the important role of PMEA in further purification of CTCs, which may yield optimal CTC culture conditions. 

Although they are not abundant, cultured CTCs from CRC patients could be utilized in several advanced techniques. For example, a minimal RNA-extraction system enables the extraction of RNA from a single cell, and semi-quantitative RT-PCR is possible (In Supplementary Figure S5, data in a few pancreatic cancer patients are shown). This could enable the measurement of expressions of certain molecular markers, such as the epithelial marker cytokeratin 19, mesenchymal marker plastin 3, and cancer stem markers ALDH1, CD133, and CD44. Another possibility is the performance of single-cell mutation analysis for *KRAS*, *PIC3CA*, *BRAF*, and other genes, as we reported [40]. Whole genome amplification, followed by Sanger sequencing, could enable the detection of mutations in CTCs. A spheroid-like structure or tumor-cell cluster can be picked up using an electrically powered micromanipulator (Supplementary Figure S6). Finally, we can perform comprehensive gene expression analysis using the C1^TM^ single-cell Auto Prep System (Fluidigm, South San Francisco, CA, USA) as we show a capture of a spheroid derived from a pancreatic cancer patient on the C1 device (Supplementary Figure S7). These investigations are currently underway in our laboratory. Additionally, there may be several alternative methods worth testing to identify optimal cell culture conditions. Possible variables include types of culture medium (modified ES medium, selected cocktail of growth factors, and highly concentrated serum, e.g., 20% FBS), plate type (non-treated, ultra-low adherent, Matrigel-coated, and coated with another extracellular matrix), culture period on the PMEA coat, and cell types. 

## 4. Materials and Methods

### 4.1. Cell Culture

Human CRC cell lines (HT29, HCT116, DLD-1, and SW480) were purchased from the American Type Culture Collection, and were authenticated by morphological inspection, STR profiling, and mycoplasma testing. Cells were cultured in either RPMI 1640 medium or DMEM (Nissui Pharmaceutical Co., Ltd., Tokyo, Japan) supplemented with 10% FBS, 100 U/mL penicillin, and 100 μg/mL streptomycin (Nacalai Tesque, Inc., Kyoto, Japan). Cells were cultured in a humidified incubator at 37 °C in an atmosphere containing 5% CO_2_.

### 4.2. Preparation of PMEA-Coated Chamber Slides

PMEA -coated chamber slides were provided from SUMITOMO RUBBER INDUSTRIES (Kobe, Japan). PMEA was synthesized according to previously reported methods [33]. Briefly, PMEA was synthesized via free radical polymerization using azobis(isobutyronitrile) (AIBN) as an initiator in toluene (2.5%(*w/v*) solution) at 60 °C for 7 h. PMEA (molecular weight: Mn = 15,000, Mw = 50,000) was dissolved in methanol (0.25%(*w/v*)). The PMEA solution was coated on the chamber slide. Fibronectin solution (200 μg/mL) (FUJIFILM Wako Pure Chemical Corporation, Osaka Japan) were then overlaid on the PMEA-coated slide, absorbed at 37 °C for 1 h. The prepared substrates were exposed to UV light on a clean bench overnight and stored at room temperature until use. 

### 4.3. Adhesion Test

The CRC cell lines HCT116, SW480, and HT29 were labeled with CellTracker Orange CMTMR Dye C2927 (Thermo Fisher Scientific, Waltham, MA, USA). Cell suspensions were adjusted to 100 cells per 10 μL by serial dilution, and the actual number of cells in 10 μL cell suspension was directly counted under a fluorescence microscope BZ-X800 (KEYENCE, Osaka, Japan). With orange fluorescence as an indicator, 100 cells were seeded (in triplicate) on 12-well tissue-culture-treated polystyrene (TCPS) dishes (IWAKI, Shizuoka, Japan), or on dishes coated with 0.25% (*w/v*) PMEA in methanol solution and 200 μg/mL fibronectin. Cells were incubated in standard medium at 37 °C overnight, and then washed twice with PBS. The attached cells were counted under a fluorescence microscope (BZ-X800). 

### 4.4. Spike Test

The HT29 and DLD-1 cells were labeled with CellTracker Orange CMTMR Dye C2927 (Thermo Fisher Scientific), and the cell suspension was adjusted to 500 cells per 50 μL by serial dilution. The actual number of cells in 10 μL cell suspension was directly counted under a fluorescence microscope (BZ-X800). Next, a cell suspension, containing approximately 500–700 cells, was mixed with 10 mL of venous peripheral blood from a healthy donor, and added to an OncoQuick tube (Greiner bio-one, Kremsmünster, Austria). After centrifugation at 1600× *g* for 20 min at 4 °C, the cells were washed once with PBS, washed again with advanced DMEM (Thermo Fisher Scientific), and then collected in 1 mL standard medium. 

One 200-μL-aliquot of the 1 mL cell suspension was used to count the CRC cells marked with orange fluorescence under a fluorescence microscope. Another 200-μL-aliquot was transferred into a chamber slide II (IWAKI) coated with 0.25% (*w/v*) PMEA in methanol solution and 200 μg/mL fibronectin (in duplicate). These cells were incubated in standard medium at 37 °C overnight. The next day, immunocytochemistry for EpCAM was performed, and stained tumor cells were counted under a microscope in the light field. 

### 4.5. Immunostaining

Cells were fixed with 10% formaldehyde for 10 min at room temperature. Immunocytochemistry was performed as previously reported [46]. Briefly, endogenous peroxidase activity was blocked using 0.3% (*v/v*) hydrogen peroxide plus 0.1% sodium azide (*w/v*) in distilled water, for 10 min, under light-shielded conditions. After serum-blocking, slides were incubated with anti-EpCAM/TROP1-HRP (VU-1D9; diluted 1:1000; Novus, Tokyo, Japan) for 30 min. A colorimetric assay based on the peroxidase reaction was performed using 0.02% 3,3′-diaminobenzidine tetrahydrochloride (Sigma Aldrich, St. Louis, MO, USA) in 0.05 M Tris-HCI (pH 7.6) containing 0.01% hydrogen peroxide, for 4 min. Counter-staining was performed using hematoxylin solution. 

### 4.6. Patient Background Data

From January 2018 to September 2022, blood samples were collected from 41 CRC patients (38 with stage IV, two with stage III, and one with stage II disease). Table 1 summarizes the patients’ background information, including age, gender, serum CEA and CA19-9 levels, tumor site, stage, tumor size, differentiation degree, metastatic organs, surgical resection of primary tumor, and treatment experience with chemotherapy or radiation prior to blood test for CTCs. Staging was determined according to the 8^th^ version of the UICC TNM classification of colorectal carcinoma [47]. All included participants gave informed consent, and this study was approved by the Institutional Review Board (approval number 14070).

### 4.7. Enrichment of CTCs in Blood Samples

We concentrated 10 mL of peripheral venous blood collected from patients using an OncoQuick tube. The concentrated cell fraction that potentially contained CTCs was transferred into a chamber slide II (IWAKI) coated with 0.25% (*w/v*) PMEA in methanol solution and 200 μg/mL fibronectin, and incubated at 37 °C overnight. The next day, cells were stained with anti-EpCAM antibody directly on the chamber slide, or transferred to 96-well or 24-well plates for subsequent cell culture. This procedure is summarized in Supplementary Figure S1.

### 4.8. Culture of CTCs

The next day, cells were transferred from PMEA chamber slide to Matrigel-coated culture plates. Cells in the chamber slide were washed twice with PBS, and then overlaid with Gibco TrypLE^TM^ Express (Thermo Fisher Scientific), and incubated at 37 °C for 10 min. Cells were collected in standard medium supplemented with 10% FBS, and then centrifuged at 300× *g* for 5 min. After discarding the supernatant, cells were recovered with modified ES medium [48] and seeded on Matrigel (Corning, NY, USA)-coated 24- or 96-well plates. Fresh ES medium was added every 2 or 3 days. 

The modified ES medium contained Advanced DMEM/F12 (Thermo Fisher Scientific), Penicillin-Streptomycin Solution Hybri-Max^TM^ (100 ng/mL; Sigma Aldrich, MO, USA), gentamycin sulfate solution (25 mg/mL, Wako Pure Chemical, Osaka, Japan), HEPES buffer solution (10 mM; Thermo Fisher Scientific), B-27 supplement (Thermo Fisher Scientific), bovine serum albumin (1 mg/mL; Sigma Aldrich), Glutamax (Thermo Fisher Scientific), recombinant murine Noggin (50 ng/mL; Pepro Tech, Cranbury, NJ, USA), recombinant human R-Spondin1 (500 ng/mL; R&D Systems, Minneapolis, MN, USA), recombinant murine EGF (50 ng/mL; Pepro Tech), recombinant mouse HGF protein (50 ng/mL; R&D Systems), recombinant mouse Wnt-3a protein (100 ng/mL; R&D Systems), Culture Sure Y-27632 (10 μM; Wako), and hydrocortisone (0.5 μM). 

### 4.9. Definition of CTC

Diagnosis of CTCs was made by cell culture and/or cytological examination (Supplementary Figure S1). Under microscopic observation, the cell showing an enlarged and irregular-shaped nucleus, and an increased N/C ratio, occasionally with a nucleolus, were considered CTCs, irrespective of positive staining of EpCAM. When cultured cells form spheroid-like structures or tumor-cell clusters, they are considered to be derived from CTCs. 

### 4.10. Statistical Analysis

Statistical differences were analyzed using Student’s *t* test for continuous variables and the Chi-squared test for non-continuous data including sex, tumor location, stage, complete resection of primary tumor, and application of chemotherapy or radiation. Statistical analyses were performed using JMP Pro 16.0.0 (SAS Institute Inc., Cary, NC, USA). A *p* value of <0.05 was considered significant.

## 5. Conclusions

In conclusion, here we demonstrated the clinical relevance of PMEA coating for the detection of CTCs from CRC patients. This simple and low-cost method may be useful for detecting CTCs from CRC cases, and possibly from many cancer types, as we suggest a usefulness in HCC and pancreatic cancer. Cultured CTCs would provide useful information at RNA and DNA levels, which may assist in the timely determination of appropriate therapeutic options. 

## Figures and Tables

**Table 1:** Patients’ background.

	N = 41
Age: years; median (range)	67 (2–80)
Sex: Male/Female	26/15
CEA ng/mL: median (range)	30 (2–2739)
CA19-9 U/mL: median (range)	26.2 (0.4–4349.5)
Location: Right/Left	8/33
Stage II/III/IV	1/2/38
Primary tumor Max diameter mm; median (range)	45 (20–140)
* Differentiation: tub/muc, por	31/3
Liver metastasis: yes/no	31/10
Lung metastasis: yes/no	12/29
Peritoneal dissemination: yes/no	7/34
Primary tumor resection: yes/no	30/11
Treatment experience of chemotherapy or radiation: yes/no	11/30

* tub: tubular adenocarcinoma, muc: mucinous carcinoma, por: poorly differentiated adenocarcinoma.

**Table 2:** Relationship between detection of CTC and/or growing tumor cells in cell culture and clinicopathological parameters.

	N = 41	CTC (+) and/or Cell Growth (+), N = 23	CTC (-) and Cell Growth (-) N = 18	*p* Value
Age: years; median (range)	67 (25–80)	67 (25–80)	68 (43–80)	0.64
Sex: Male/Female	26/15	15/8	11/7	0.79
CEA ng/mL: median (range)	30 (2–2739)	21 (2–2739)	33.5 (2–1956)	0.89
CA19-9 U/mL: median (range)	26.2 (0.4–4349.5)	26.2 (0.4–2870.2)	26.55 (0.4–4339.5)	0.82
Location: Right/Left	8/33	5/18	3/15	0.68
* Differentiation: tub/muc, por	31/3	17/3	14/0	0.07
Primary tumor resection: yes/no	30/11	17/6	13/5	0.90
Chemotherapy or radiation: yes/no	11/30	3/20	8/10	0.02

* tub: tubular adenocarcinoma, muc: mucinous carcinoma, por: poorly differentiated adenocarcinoma.

## Data Availability

Not applicable.

## Supplementary Materials

The following supporting information can be downloaded at: https://www.mdpi.com/article/10.3390/ijms24043949/s1.
