# Supplementary material for: Simple Detection and Culture of Circulating Tumor Cells from Colorectal Cancer Patients Using Poly(2-Methoxyethyl Acrylate)-Coated Plates
Source: Int J Mol Sci. 2023 Feb 16;24(4):3949. doi: 10.3390/ijms24043949 (PMC9959032; doi:10.3390/ijms24043949)
Supplement: Supplementary file 1 [file ijms-24-03949-s001.zip › ijms-2167426-supplementary.pdf]

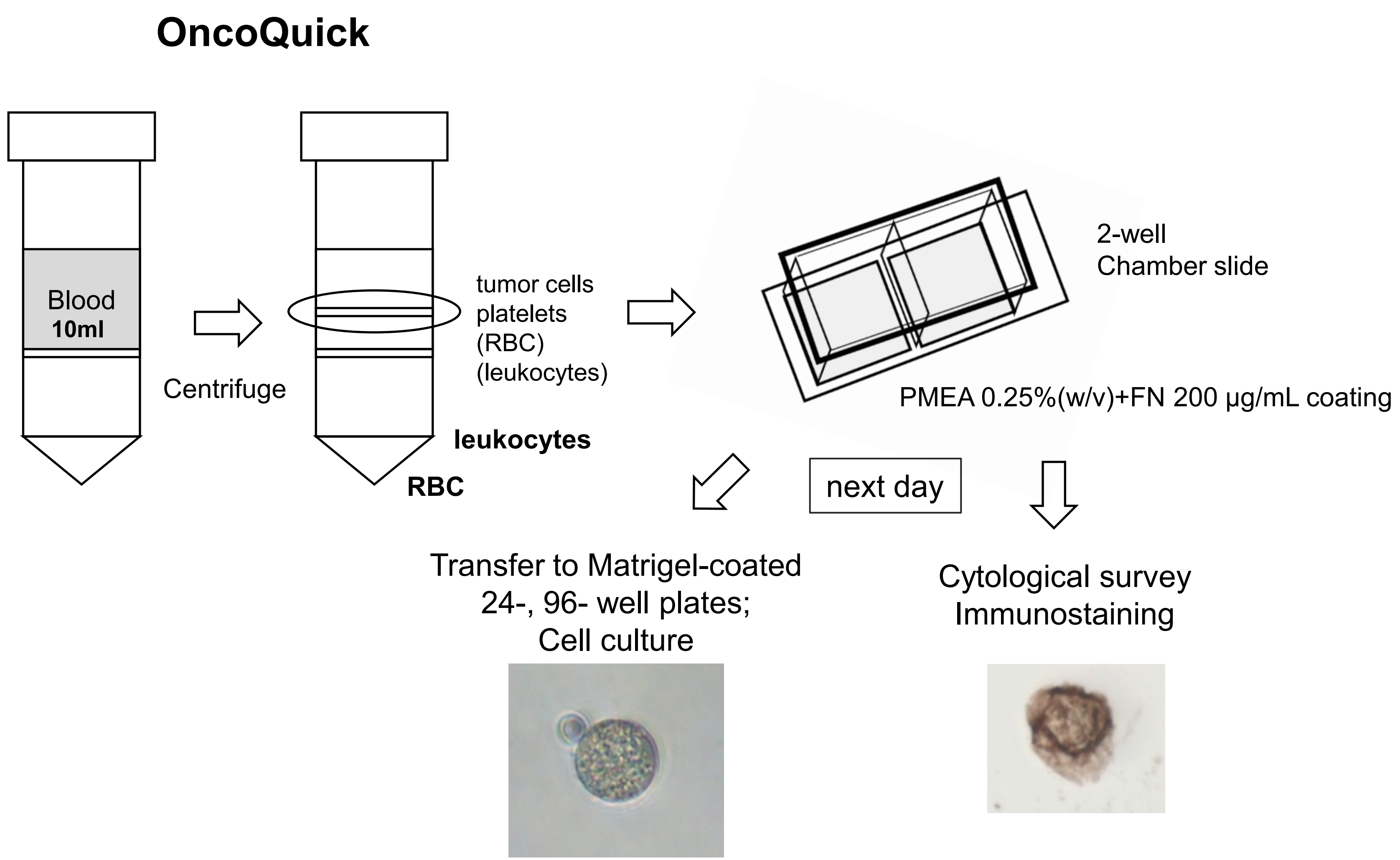

Fig. S1. Scheme of CTC collection and analysis. OncoQuick tube and PMEA were used for enrichment of CTCs. Cells were incubated overnight on the PMEA/FN-coated slides. The next day, more than half fraction was transferred to Matrigel-coated plates. The remaining cells were subject to immunostaining for EpCAM. FN: fibronectin.

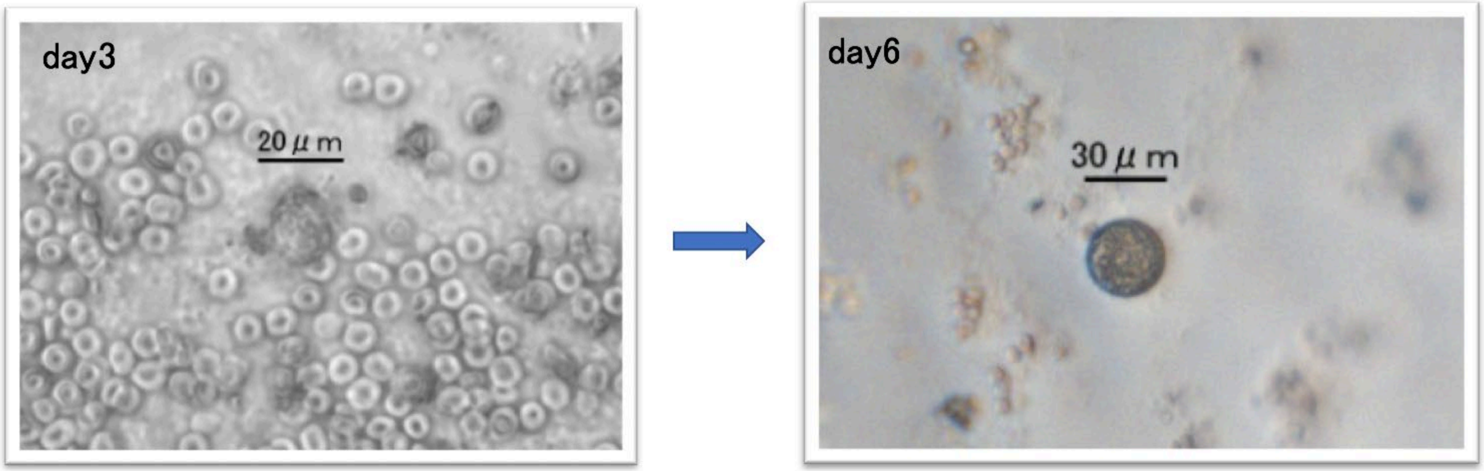

Fig. S2. Culture of CTC in Case #33. A spheroid-like structure grew up from day 3 to day 6 while many red blood cells and some leukocytes disappeared during the period.

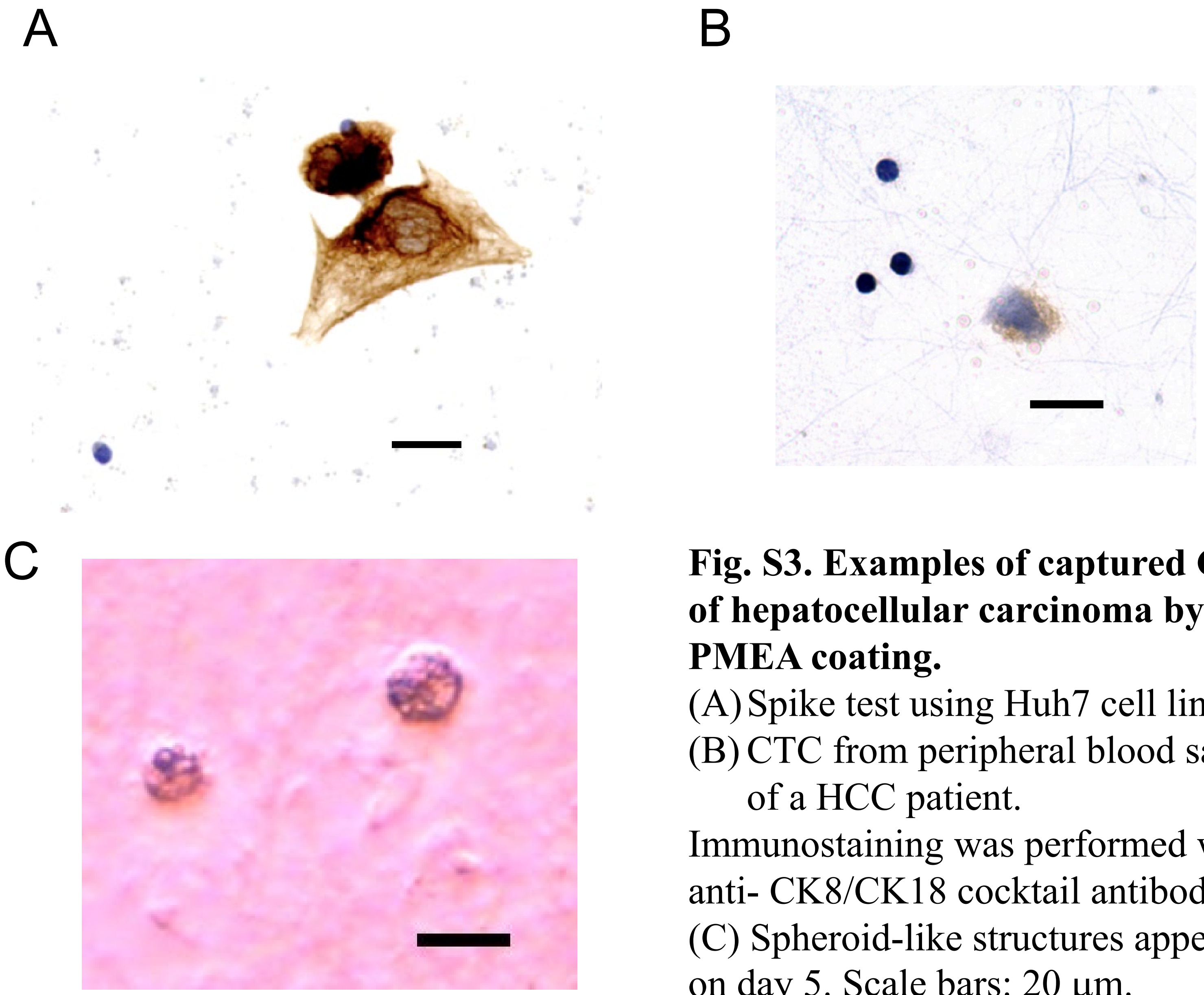

Fig. S3. Examples of captured CTCs of hepatocellular carcinoma by PMEA coating.

(A) Spike test using Huh7 cell line.

(B) CTC from peripheral blood sample of a HCC patient.

Immunostaining was performed with anti- CK8/CK18 cocktail antibody.

(C) Spheroid-like structures appeared on day 5. Scale bars: 20 μm.

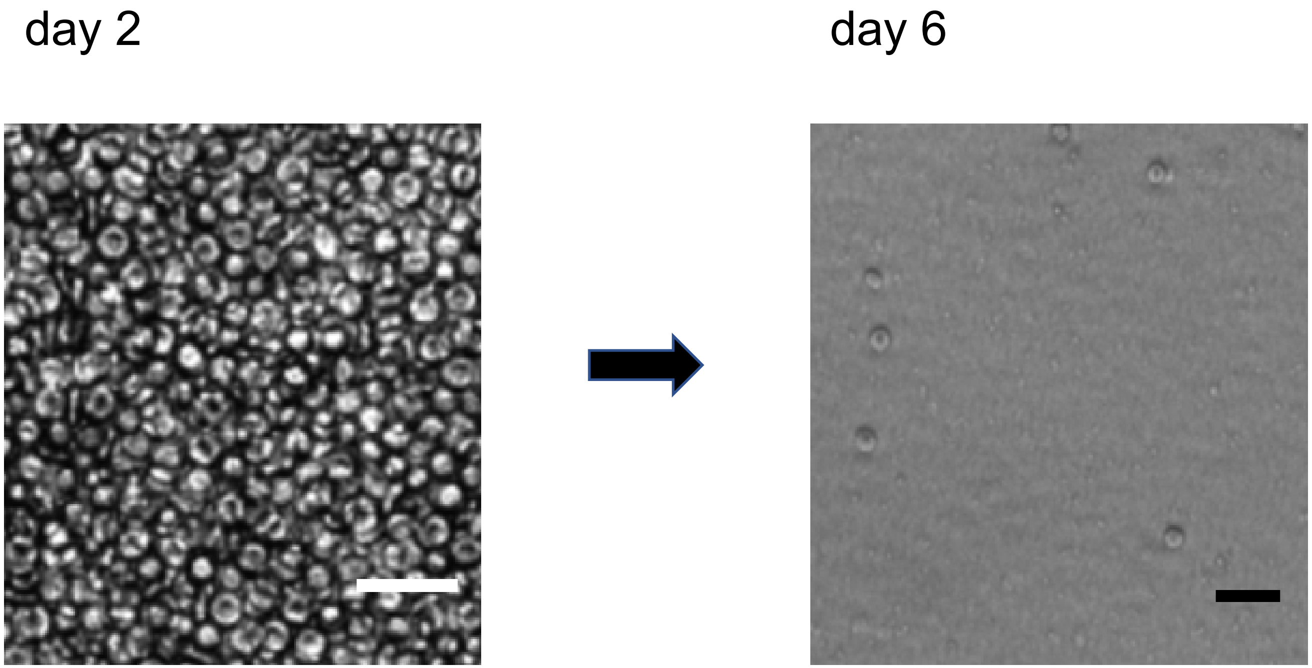

Fig. S4. A direct culture after concentration with the OncoQuick tube. Numerous red blood cells and leukocytes occupied a chamber slide on day 2. On day 6, cells mostly disappeared.

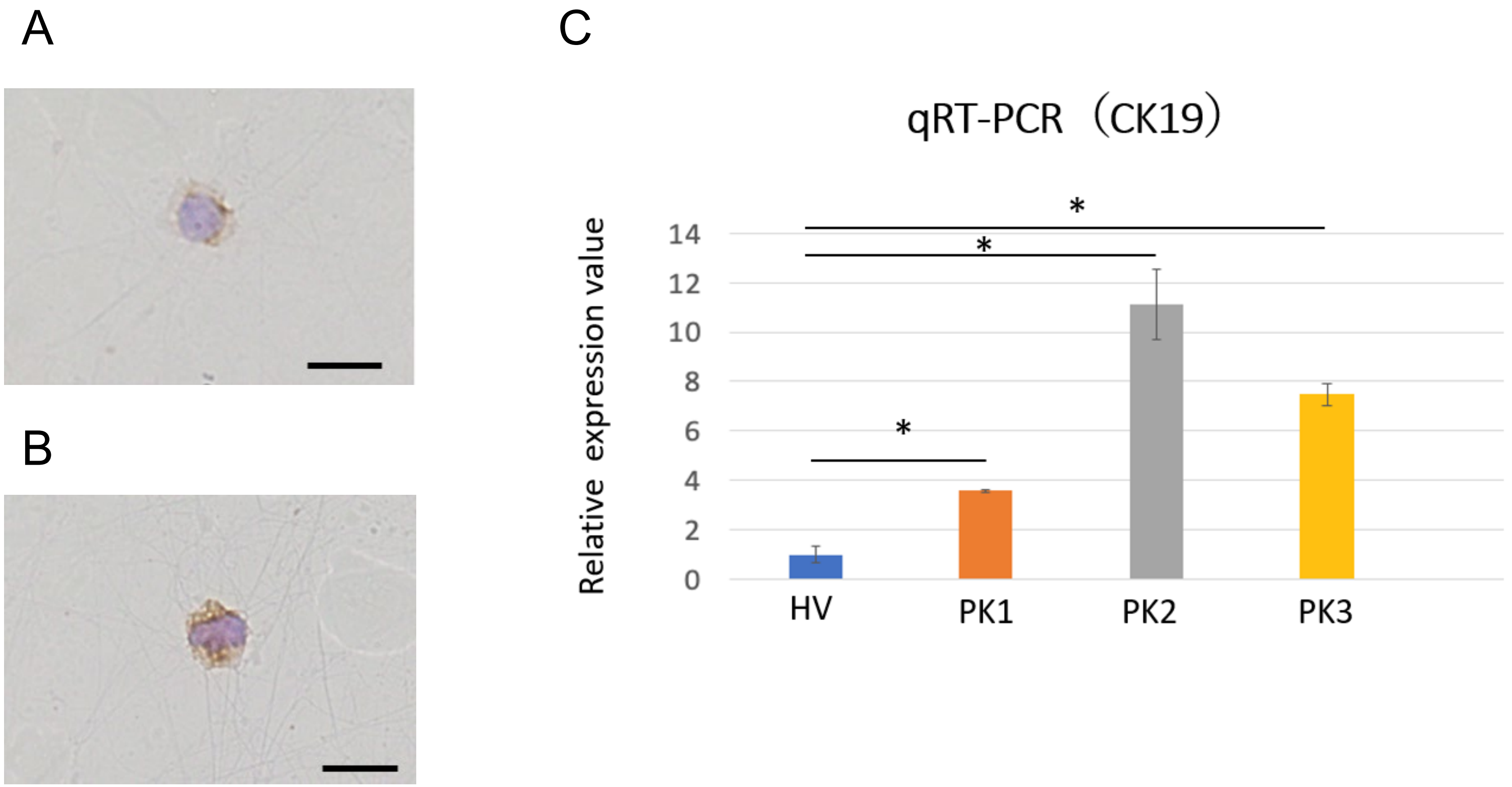

Fig. S5. Examples of captured CTCs of pancreatic carcinoma by PMEA coating.
(A) A CTC from portal vein blood sample of a pancreatic cancer patient.
(B) A CTC from portal vein blood sample of another pancreatic cancer patient.
Immunostaining was performed with anti-CK19 antibody. Scale bars: 20 μm.
(C) qRT-PCR for CK19 mRNA expression. Cultured cells from three patients (PK1, PK2, PK3) were collected on day7. Blood sample from a healthy volunteer (HV) served as negative control. *P<0.05.

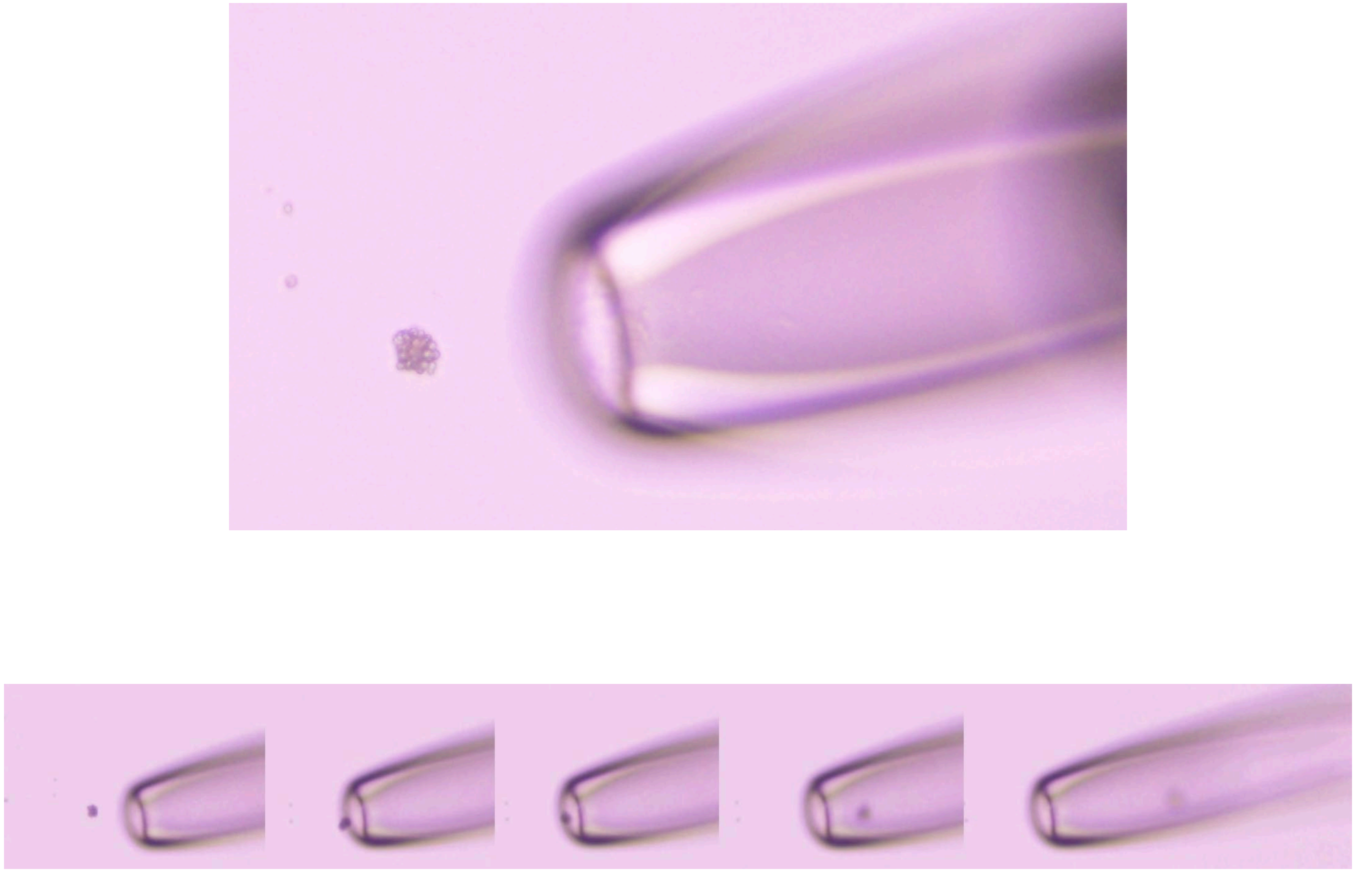

Fig. S6. The electrically-powered micromanipulator can pick up a cluster of tumor cells.

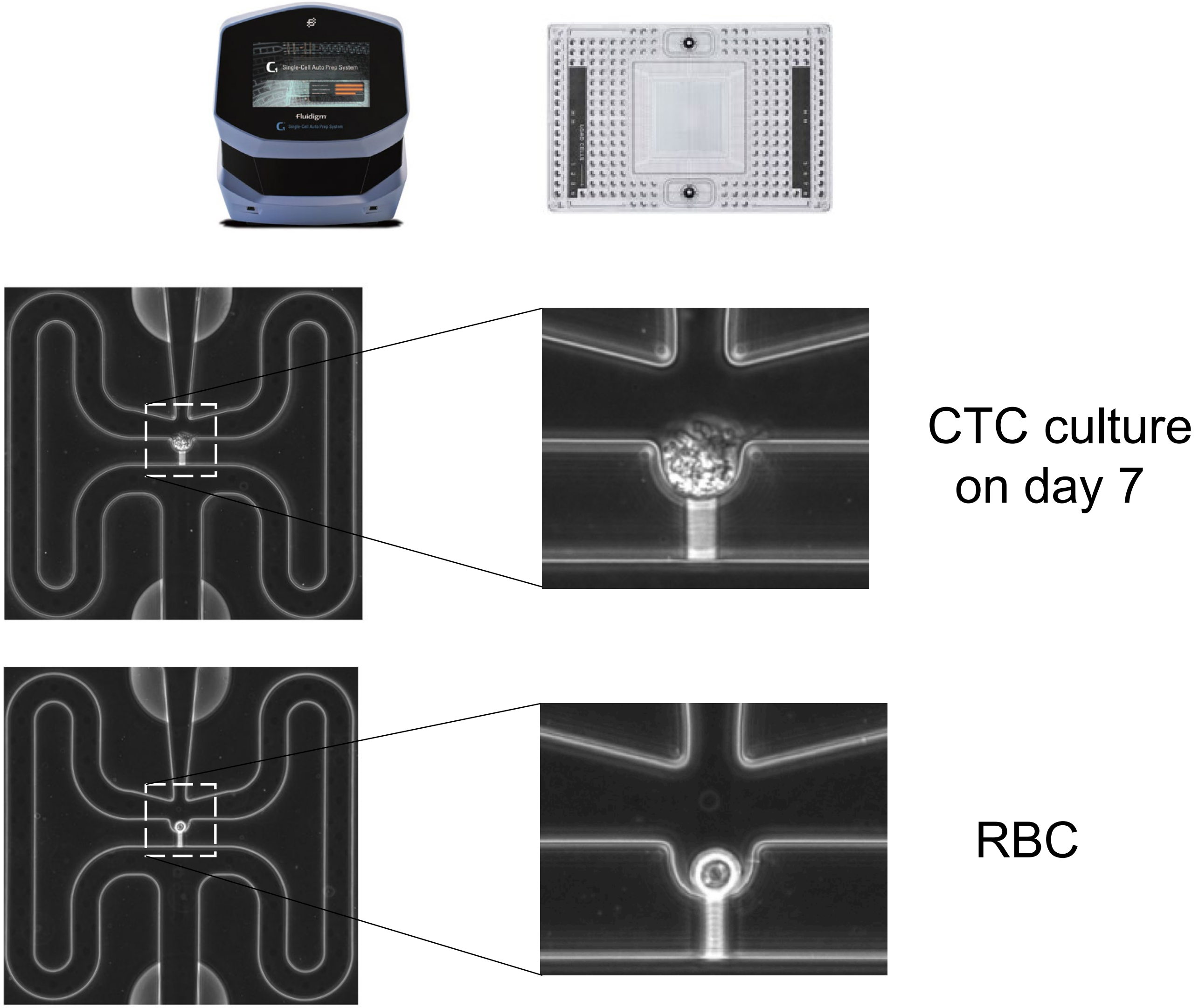

Fig. S7. Comprehensive gene expression analysis using C1™ single cell Auto Prep System (Fluidigm). A spheroid derived from CTC of a pancreatic cancer patient was captured in a cell deposit space on day 7. A red blood cell (8 μm in diameter) was also captured.

Table S1. Recovery rate of CRC cells from blood sample

HT29

Initial cell number/10μL	After OncoQuick concentration		ICC (+)	Recovery rate
148	132 (89.2%)	Sample 1	98	74.2% (98/148)
		Sample 2	103	78.0% (103/148)

DLD-1

Initial cell number/10μL	After OncoQuick concentration		ICC (+)	Recovery rate
121	110 (90.9%)	Sample 1	87	79.1% (87/121)
		Sample 2	83	75.5% (83/121)

ICC: Immunocytochemistry

Table S2. Relationship between detection of circulating tumor cells and clinicopathological parameters

	N = 41	CTC (+), N = 18	CTC (-), N = 23	P value
Age: years; median (range)	67（25-80）	68.5 (40-80)	67 (25-80)	0.73
Sex: Male /Female	26/15	13/5	13/10	0.30
CEA ng/mL; median (range))	30 (2-2739)	13.75 (2-2739)	50 (2-1956)	0.89
CA19-9 U/mL; median (range))	26.2 (0.4-4349.5)	17.5 (0.4-2870.2)	36 (0.4-4339.5)	0.61
Location: Right / Left	8/33	4/14	4/19	0.70
*Differentiation : tub / muc, por	31/3	12/3	19/0	0.02
Primary tumor resection : yes / no	30/11	12/6	18/5	0.41
Chemotherapy or radiation : yes /no	11/30	3/15	8/15	0.19

* tub: tubular adenocarcinoma, muc: mucinous carcinoma, por: poorly differentiated adenocarcinoma

Table S3. Relationship between presence of growing tumor cells in cell culture and clinicopathological parameters

	N = 33	Culture (+) N = 18	Culture (-) N = 15	P value
Age: years; median (range)	70 (25-80)	70 (25-80)	69 (43-80)	0.52
Sex: Male / Female	20/13	12/6	8/7	0.44
CEA ng/mL; median (range))	30 (2-2739)	23.55 (2-1956)	30 (2-2739)	0.96
CA19-9 U/mL; median (range))	26.2 (0.4-4349.5)	22.6 (0.4-1691.8)	28.6 (0.4-4339.5)	0.43
Location: Right / Left	8/25	3/15	5/10	0.27
*Differentiation : tub / muc, por	26/2	15/2	11/0	0.15
Primary tumor resection : yes / no	25/8	14/4	11/4	0.77
Chemotherapy or radiation : yes / no	10/23	1/17	9/6	0.0004

* tub: tubular adenocarcinoma, muc: mucinous carcinoma, por: poorly differentiated adenocarcinoma
